# DMD-based hyperspectral microscopy with flexible multiline parallel scanning

**DOI:** 10.1038/s41378-021-00299-2

**Published:** 2021-09-01

**Authors:** Xue Dong, Geng Tong, Xuankun Song, Xingchen Xiao, Yiting Yu

**Affiliations:** 1 Research & Development Institute of Northwestern Polytechnical University in Shenzhen, Shenzhen, 518057 China; 2 Ningbo Institute of Northwestern Polytechnical University, Ningbo, 315103 China; 3 Key Laboratory of Micro/Nano Systems for Aerospace (Ministry of Education), Northwestern Polytechnical University, Xi’an, 710072 China; 4 Shaanxi Province Key Laboratory of Micro and Nano Electro-Mechanical Systems, Northwestern Polytechnical University, Xi’an, 710072 China

**Keywords:** Micro-optics, Optical sensors

## Abstract

As one of the most common hyperspectral microscopy (HSM) techniques, line-scanning HSM is currently utilized in many fields. However, its scanning efficiency is still considered to be inadequate since many biological and chemical processes occur too rapidly to be captured. Accordingly, in this work, a digital micromirror device (DMD) based on microelectromechanical systems (MEMS) is utilized to demonstrate a flexible multiline scanning HSM system. To the best of our knowledge, this is the first line-scanning HSM system in which the number of scanning lines *N* can be tuned by simply changing the DMD’s parallel scanning units according to diverse applications. This brilliant strategy of effortless adjustability relies only on on-chip scanning methods and totally exploits the benefits of parallelization, aiming to achieve nearly an *N*-time improvement in the detection efficiency and an *N*-time decrease in the scanning time and data volume compared with the single-line method under the same operating conditions. To validate this, we selected a few samples of different spectral wavebands to perform reflection imaging, transmission imaging, and fluorescence imaging with varying numbers of scanning lines. The results show the great potential of our DMD-based HSM system for the rapid development of cellular biology, material analysis, and so on. In addition, its on-chip scanning process eliminates the inherent microscopic architecture, making the whole system compact, lightweight, portable, and not subject to site constraints.

## Introduction

As a powerful detection tool, hyperspectral imaging combines imaging and spectroscopy technologies and is used in numerous areas, such as remote sensing, agricultural monitoring, and arch eology^1–5^. Recently, its application domain has been extended to microsamples, which has given rise to a new name, i.e., hyperspectral microscopy (HSM)^6^. Due to the ability to capture a sample’s three-dimensional (3D) data cube with two-dimensional (2D) spatial information and one-dimensional (1D) spectral information, HSM has been explored for various applications, including diagnosis of tumor tissue^7,8^, analysis of chemical and biological materials^9–11^, and visualization of cells and embryos^12–15^.

Based on the acquisition methods of the data cube, the existing implementations of HSM can be divided into four major categories: point-scanning HSM, line-scanning HSM, wavelength-scanning HSM, and snapshot HSM^3,16–18^. The wavelength-scanning mode is time-consuming, and compromises must be made between the spatial and spectral performance^2,19^. Among these methods, snapshot HSM is believed to give the shortest acquisition time^6^, but it needs to trade-off spatial and spectral resolutions to accommodate all the spatial and spectral data simultaneously, making it difficult to provide high spectral sampling^20–22^. Relatively speaking, the point-scanning and line-scanning modes perform well at both spatial and spectral resolutions^2^. However, the slow point-scanning method takes such a long time that it is unsuitable for real-time analysis and large samples. In contrast, the line-scanning method collects the one-line spectra of a sample at one time, thereby generating a higher acquisition speed. Based on this, many types of commercial microscopes, such as light-sheet microscopes and confocal microscopes, have been developed as line-scanning HSM^6,13,23^. Nevertheless, the acquisition speed of line-scanning HSM is still considered to be insufficiently fast, making the detection of rapid biological and chemical processes difficult. In addition, some biological samples are fragile and easily cause failure.

More recently, the parallel strategy has been applied to traditional point-scanning and line-scanning approaches to further improve the imaging efficiency. In 2015, gigapixel multispectral microscopy was presented to record large numbers of point spectra on a sample simultaneously with a microlens array, equivalent to massively parallelizing point-scanning systems^24^. Although the scanning time is efficiently reduced, the simultaneous acquisition of the spatial and spectral information of too many points leads to a low spectral resolution of dozens of nanometers. In 2017, a parallelized microscopy technique was reported, enabling fast and light-efficient 3D imaging by parallelizing the illumination scheme with three light sheets^25^. In 2019, a variant of confocal microscopy achieved multiplane parallel imaging by simultaneous scanning of several pinholes located at different axial depths^26^. Later, a parallel scanning imaging approach was proposed to make multipoint superresolution imaging possible. The key point of this approach is that it combines standard microscopy with a microlens array made of multiple barium titanate glass microspheres and polydimethylsiloxane^27^. Clearly, the brilliant strategy of easily achieving multipoint or multiline parallelized imaging can shorten the scanning time to some extent. However, the number of scanning units in existing microscopy systems is always fixed for any application, restricted by the conventional scanning mechanism, such as piezoelectric actuators or scanning mirrors. Hence, further improvement in the imaging efficiency is limited. To date, no line-scanning HSM has been made available to flexibly offer a variable number of scanning lines when faced with various samples exhibiting distinct spectral signatures. In addition, either the traditional HSM instruments or variants thereof are modified based on bulky microscope platforms, which are highly suitable for laboratories and hospitals but not portable on-site cases.

Here, inspired by the high-efficiency parallel strategy and microelectromechanical systems (MEMS), we introduce a parallelized line-scanning HSM scheme with a digital micromirror device (DMD) to obtain variable scanning lines according to the object’s spectral bandwidth, and we call it DMD-based flexible multiline parallel scanning hyperspectral microscopy (DFMLPS-HSM). In fact, taking advantage of its fast refresh rate of thousands of Hertz, the DMD is usually used as a binary configurable mask for 3D random-access microscopic imaging and snapshot spectral imaging^28–32^. Unlike the reported results, we employ the DMD as a smart multiline controllable scanner on the chip scale behaving like the parallel scanning of several slits. Previously, we established a DMD-based hyperspectral imaging system with adjustable spatial and spectral resolutions for macrotargets and demonstrated three types of scanning modes, rough scanning, fine scanning, and regional scanning^33^. Based on this, this paper extends the multiline scanning capability to realize HSM for microsamples. In addition, the number of scanning lines is alterable by easily changing the number of DMD parallel scanning units. This flexibility allows the DFMLPS-HSM to always provide the optimal number of scanning lines for different samples and applications, thereby significantly reducing the acquisition time, improving the detection efficiency, and compressing the amount of data. In addition, the DFMLPS-HSM system is free from the inherent microscopic architecture, which contributes to a great reduction in the weight and dimensions. Therefore, the whole system has the characteristics of compactness and portability and is not confined to particular sites.

## Results and discussion

### System setup

**Fig. 1:**
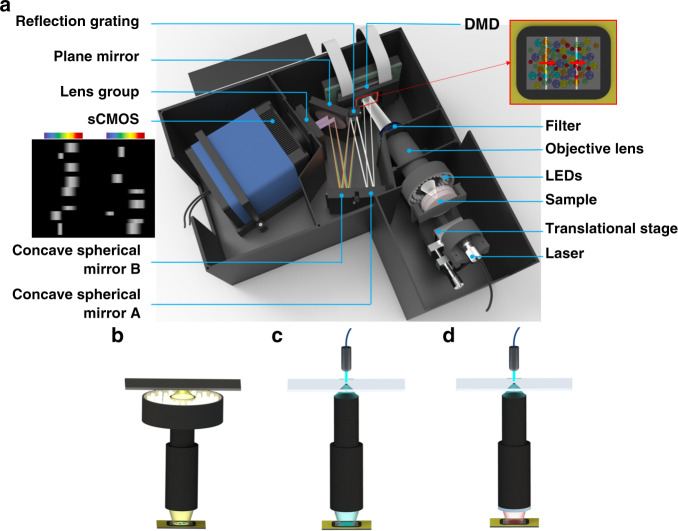
System setup. **a** 3D model and optical path without the top cover. The schematics of the imaging subsystem configurations corresponding to three different imaging modes are shown: **b** reflection imaging with a ring of white LEDs and a longpass filter, **c** transmission imaging with a laser, and **d** fluorescence imaging with a laser and a notch filter

The 3D model of the DFMLPS-HSM system with dimensions of 27 cm (W) × 27 cm (D) × 13 cm (H) is illustrated in Fig. 1a, consisting of two major subsystems, i.e., the imaging subsystem and spectral dispersion subsystem. The former is composed of a light module including a laser and a ring of white LEDs with uniform light, a sample, a manual translational stage, a 10× customized objective lens, a filter, and a DMD (DLP7000) with a matrix of 1024 × 768 individually addressable micromirrors. The latter is composed of two concave spherical mirrors (A and B, *f* = 300 mm), a reflection grating with a groove density of 600 l/mm, a plane mirror, a lens group, and an sCMOS (Pco.edge 4.2bi) with a matrix of 2048 × 2048 pixels. All the optical fixtures and the outer framework of the prototype are 3D printed and made of black ABS plastics or nylon. Some of them are covered with flexible light absorbers to reduce the impact of stray light on image quality. The field of view (FOV) of the DFMLPS-HSM system is ~1 mm, and the working waveband covers the visible range from 450 to 700 nm.

The working principle is summarized as follows: First, we manually adjust the translational stage to clearly focus the sample onto the DMD via the objective lens, and the formed 2D image is thereby divided into 1024 columns by the DMD micromirrors. Then, the DMD functions as a multiline scanner, simultaneously reflecting a partial image by *N* discrete columns to the spectral dispersion subsystem each time, which can be achieved by electrically turning on *N* columns of micromirrors at the same time. The image generated thereby, composed of *N* discrete fragments, is successively collimated by the concave spherical mirror A and then dispersed by the reflection grating. The resulting *N* spectra, which do not overlap in space, are focused onto the sCMOS by the concave spherical mirror B and the lens group and are finally recorded as a spectrally dispersed image. As the grouped micromirrors of the *N* columns rotate simultaneously and scan continuously along the direction of the red arrow (Fig. 1a), the processed partial image is also changed. After the spectrally dispersed image of the last partial image, also reflected by *N* columns, is acquired, the DFMLPS-HSM system completes the whole multiline scanning process and obtains the sample’s 3D data cube. The sequential selection of *N* column images implemented by the DMD is similar to parallelizing several line-scanning HSM systems based on their corresponding traditional slits.

To realize the versatile DFMLPS-HSM system, three imaging modes are offered to end users, i.e., reflection imaging, transmission imaging, and fluorescence imaging, as shown in Fig. 1b–d. The removable elements in the imaging subsystem, including the light module and the filter, are available for the realization of different imaging modes. The illumination provided by the ring of white LEDs and the longpass filter directly in front of the objective lens enable reflection imaging. The light intensity of the white LEDs is adjustable, and the working waveband of the longpass filter is selected to be the same as that of the system. A laser with a narrow bandwidth is chosen for illumination to perform rapid transmission imaging of some transparent samples. Furthermore, an appropriate notch filter is applied in place of the longpass filter to filter out the transmitted laser light and to complete the sample’s fluorescence imaging. The notch filter can be replaceable according to the wavelength range of the laser used.

### Performance test

**Fig. 2:**
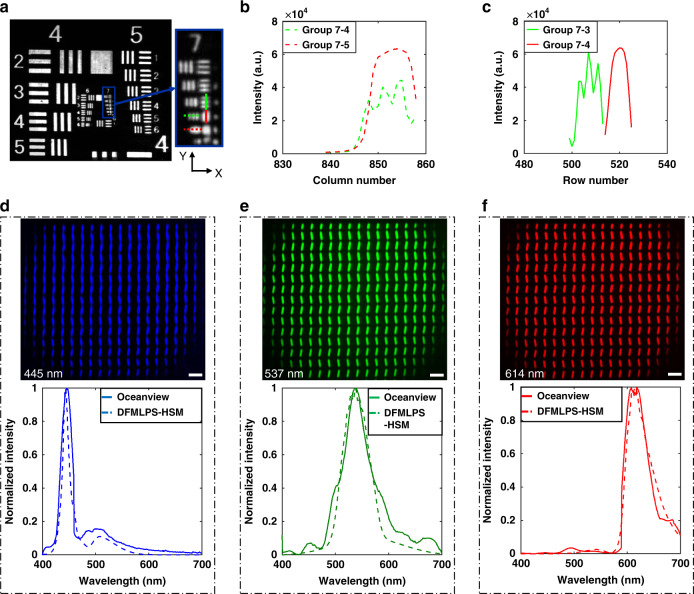
System performance test of spatial resolution and spectral measurement. **a** USAF resolution target’s grayscale spectral image in the central wavelength of 491.7 nm, as well as its partially magnified view. **b** Vertical line profiles of group 7-4 and group 7-5. **c** Horizontal line profiles of group 7-3 and group 7-4. Spectral images and spectra obtained by the DFMLPS-HSM prototype and a commercial spectrometer called Oceanview of a mobile phone screen displaying three colors: **d** blue, **e** green, and **f** red. Scale bars: 100 µm

Through the transmission imaging of a United States Air Force (USAF) resolution target with a 488 nm laser, we first evaluated the system’s spatial resolution. As the laser’s spectral bandwidth was from *λ*_1_ = 486.7 nm to *λ*_2_ = 496.7 nm, the number of DMD parallel scanning lines *N* *=* 15, and thus, the starting scanning columns *x*_*i*_ = 71*i* − 70 (*i*∈[1, 15]) were calculated according to Eq. (10) and Eq. (14) in the “Materials and methods” section. It is almost impossible for conventional line-scanning HSM instruments to obtain so many scanning lines by mechanical motions or system modification, which reveals the merits of MEMS technology in terms of small volume, fast response, and ease of integration. Moreover, the shortest exposure time *t* = 6 ms was set as a consequence of the strong laser intensity, achieving the scanning time *t*_*m*_ = 426 ms calculated by Eq. (15). From the results in Fig. 2a, it can be seen that there is no significant image distortion in the entire FOV, showing the good imaging quality given by the DFMLPS-HSM prototype. It has the ability to distinguish the lines in group 7-4 (Fig. 2b, linewidth = 2.76 µm) in the *X* direction and those in group 7-3 (Fig. 2c, linewidth = 3.1 µm) in the *Y* direction.

Next, a mobile phone screen was selected as the target to verify the validity of the DFMLPS-HSM prototype’s spectral measurement. The mobile phone screen was controlled to successively display three colors, blue, green, and red, and we measured the spectra of each screen state with our prototype and a commercial spectrometer called Oceanview. In addition, the spectral images of three peak wavelengths corresponding to the three screen states were also acquired thanks to the imaging ability of the DFMLPS-HSM. The results in Fig. 2d–f show that great consistency can be obtained, proving the validity of the DFMLPS-HSM prototype.

### Transmission imaging and fluorescence imaging of HeLa cells

To further demonstrate the DFMLPS-HSM system’s ability to detect biological specimens, images of HeLa cells labeled with InP@ZnS quantum dots were acquired by conducting transmission bright-field imaging and fluorescence imaging. A 488 nm laser was chosen to provide transillumination and excitation-wavelength light. In the case of HeLa cell transmission imaging, the scanning parameters, including the exposure time and the number of scanning lines, were the same as those of the abovementioned USAF resolution target. After transmission imaging, we prepared fluorescence images by inserting a 488 nm notch filter in front of the objective lens to block the excitation-wavelength light and setting the scanning parameters. Since the emitted wavelength of the InP@ZnS quantum dots covered approximately *λ*_1_ = 620 nm to *λ*_2_ = 700 nm^34^, the number of DMD parallel scanning units *N* *=* 2, and thus, the starting scanning columns *x*_*i*_ = 557*i* − 556 (*i*∈[1, 2]) were given according to Eqs. (10) and (11) in the “Materials and methods” section. For traditional line-scanning HSM instruments, it is difficult to achieve double-line parallelized imaging without increasing the system complexity. In addition, a long exposure time of 150 ms was selected because of the weak fluorescence signals, resulting in the whole scanning time being ~1.4 min as calculated by Eq. (12). Furthermore, we performed pseudocolor and fusion processing on the resulting images for better visualization.

**Fig. 3:**
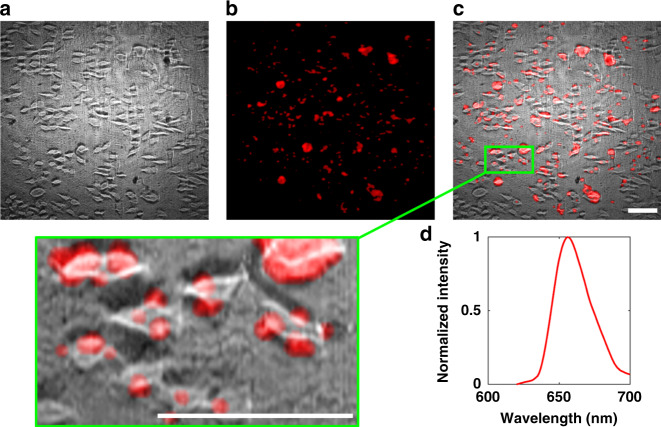
Transmission imaging and fluorescence imaging of HeLa cells. **a** Transmission image; **b** false-color fluorescence image; **c** fused image and its partially magnified view in the green box; **d** fluorescence emission spectra. Scale bars: 200 µm

Figure 3 presents the results of the transmission image, the fluorescence image, and the emission spectra of the HeLa cells. As shown in Fig. 3c, the cellular outlines are clearly visible, especially in the enlarged view, which is highlighted with a green box. By comparison, the fluorescence image is blurry because the system’s spatial resolution is not high enough to observe the HeLa cells’ internal structures dyed by the InP@ZnS quantum dots. Nevertheless, the DFMLPS-HSM system has still shown its ability to reveal the general distribution, morphology, and spectral characteristics of biological specimens and may find potential applications, such as quickly differentiating between normal cells and diseased cells.

### Reflection imaging of colored microspheres

To verify the HSM ability of the DFMLPS-HSM system in the full working waveband (*λ*_1_ = 450 nm and *λ*_2_ = 700 nm), we implemented reflection imaging of five kinds of monodisperse polystyrene microspheres with different colors (blue, green, yellow, orange, and red). The size of the blue microspheres was ~65 μm, and the size of the other microspheres was ~35 μm. These five microsphere solutions with the same concentration of 2% (wt/vol) were mixed together thoroughly and then distributed on a silicon chip. After the calculation based on the “Materials and methods” section, we carried out single-line scanning, i.e., *N* *=* 1. To give the sCMOS just enough time to record the spectrally dispersed images with the desired quality, the correct exposure time of 10 ms was set, and it took ~10 s to complete the whole scanning process.

**Fig. 4:**
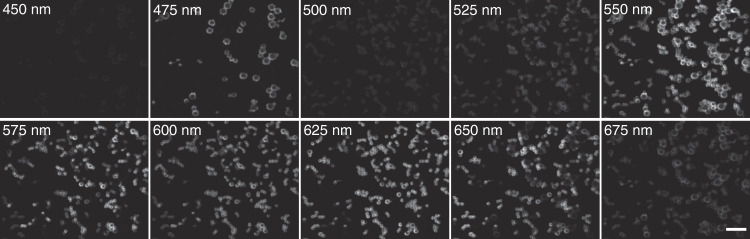
Colored microsphere grayscale spectral images of ten channels. Scale bars: 200 µm

**Fig. 5:**
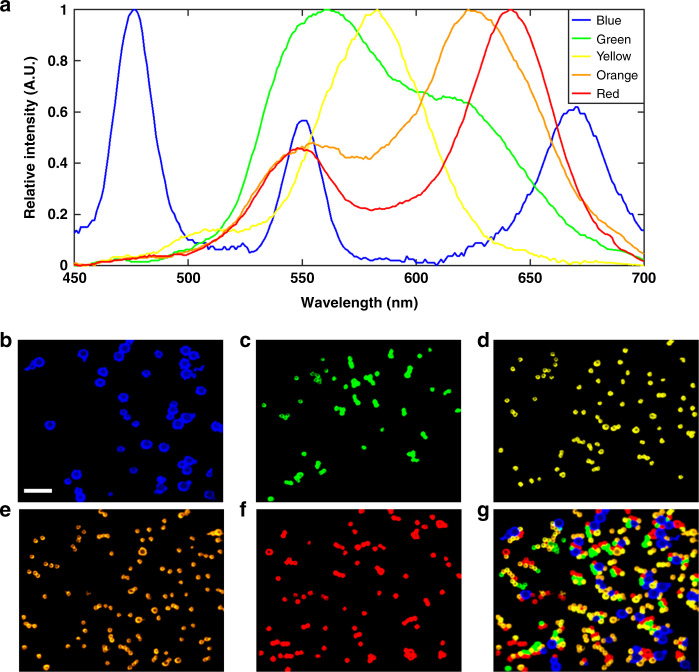
Spectra and linear unmixing of five kinds of colored microspheres. **a** Spectral curves of five kinds of colored microspheres. **b**–**f** Unmixed false-colored images of each colored microsphere. **g** False-colored fused image of five kinds of colored microspheres. Scale bars: 200 µm

The resulting data cube was downsampled from 1250 spectral channels to 10 channels for convenience, producing ten grayscale spectral images, as shown in Fig. 4. By simply observing the spectral images, it was difficult to find the positions of each kind of colored microsphere even though there are intensity changes between different spectral images. For this reason, we manually identified some regions of interest and calculated their average spectra to determine five spectral end members (Fig. 5a). Then, we applied the linear unmixing method to successfully separate these five kinds of colored microspheres and obtained unmixed false-colored images of each kind of colored microsphere (Fig. 5b–f) by implementing pseudocolor processing. Summing over the five unmixed images yielded a fused image, as shown in Fig. 5g. The recognition accuracy of the green, yellow, orange, and red microspheres was 95%, 94%, 88%, and 92%, respectively, while the recognition accuracy of the blue microspheres was not sufficiently high and reached only 81%. The reason may be that the blue microspheres are larger and prone to blocking and defocusing phenomenon. Here, unmixing the colored microspheres is considered to be successful when the recognition accuracy of each colored microsphere is more than 80%. The results indicate that the DFMLPS-HSM system has the capabilities of detection and classification, which may be used in drug screening and material analysis. Although the excessively broad spectral bandwidth makes it difficult to perform multiline parallel scanning, future improvements in selecting a detector with a larger array size will address this problem.

### Conclusion

In this paper, the DFMLPS-HSM system is proposed by introducing a smart chip, a DMD, as an ideal multiline scanner to take full advantage of parallelization due to its small size, fast response, and so on, and a first-generation prototype is established. Briefly, the DFMLPS-HSM system employs the DMD to parallelize several common single-line scanning HSM systems. Under the same conditions, it provides nearly an *N*-time improvement in speed and an *N*-time decrease in data volume compared with the single-line method. To the best of our knowledge, this is the first time that multiline parallel scanning HSM has allowed the number of scanning lines to be tuned, and a simple method for setting the number of scanning lines is also given. We tested its performance in reflection imaging, transmission imaging, and fluorescence imaging by choosing different samples and DMD parallel scanning lines. The advantages of high scanning efficiency and flexibility in changing the number of scanning lines make the system suitable for fast hyperspectral imaging of diverse varieties of microsamples with different wavebands. Moreover, the system’s compactness and portability help in on-site analysis, especially for urgent detection in remote regions. Our future plan is to greatly increase the system’s spatial resolution by using alternative objective lenses with higher magnifications. On the other hand, a detector with a larger array size and a shorter exposure time will be utilized to further improve the system’s working waveband and scanning efficiency in the next generation.

## Materials and methods

### Multiline parallel scanning of the DMD

According to the approximate spectral range of the sample and the law of spectral shift on the sCMOS caused by the DMD’s single-line scanning, the number of DMD parallel scanning lines *N* can be calculated.

**Fig. 6:**
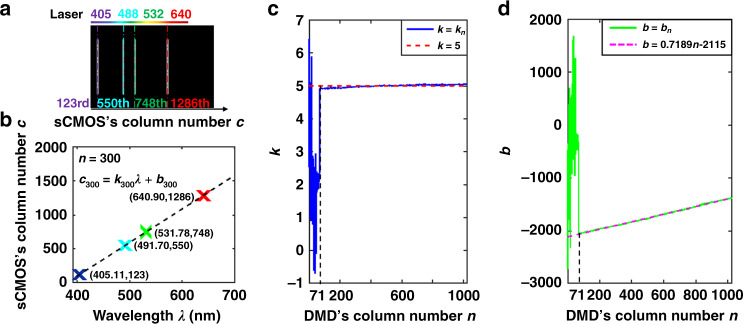
Spectrum calibration. **a** Spectrally dispersed image of the four lasers caused by the deflection of the 300th-column micromirrors. **b** The fitting result of the 300th dispersive equation. Comparison of *k* (**c**) and *b* (**d**) of the fitting result and the measured data

Through the spectrum calibration experiment with four lasers whose central wavelengths are known (405.11, 491.70, 531.78, and 640.90 nm), we found a linear relation between the central wavelength *λ* and the sCMOS column number *c* by analyzing their experimental data and thus obtained 1024 dispersive equations corresponding to the 1024 columns of micromirrors of the DMD. Taking the 300th-column micromirrors as an example, the sCMOS collected its corresponding spectrally dispersed image, as shown in Fig. 6a. The positions of the four lasers’ central wavelengths on the sCMOS were given by Gaussian fitting, and they were the 123rd, 550th, 748th, and 1286th positions. Fig. 6b depicts a linear relationship between the central wavelength *λ* and the sCMOS column number *c*_300_, and the 300th dispersive equation is given by Eq. (1). Note that the sCMOS column number must be a positive integer, and therefore, it needs to be rounded up. After spectrum calibration, 1024 dispersive equations with known slopes and intercepts were obtained. Assuming that the DMD column number is *n* (*n*∈[1, 1024]) and that each known dispersive equation’s slope and intercept are represented as *k*_*n*_ and *b*_*n*_, respectively, all the dispersive equations can be represented as in Eq. (2). Furthermore, analyses were conducted on the 1024 slopes and 1024 intercepts. The results indicate that the changes in most slopes are minor except for the first 70 slopes (Fig. 6c), and the mathematical relation between the DMD column number *n* and the intercept *b*_*n*_ is linear except for the first 70 intercepts (Fig. 6d). Since some laser light is blocked by the edges of the optical fixtures in the spectrum calibration experiment, the sCMOS cannot record the full dispersed spectrum of the lasers, resulting in the error of the first 70 dispersive equations. In fact, the number of DMD columns is sufficient to scan the sample’s image in the desired field of view and construct the spectral images we need. Therefore, sacrificing a few columns is acceptable to achieve a compact system prototype. For convenience in calculation, the slope *k*_*n*_ of the *n*th dispersive equation is replaced by the mean value $\bar k$ = 5 acquired by Eq. (3), and the relation between the DMD column number *n* and the dispersive equation intercept *b*_*n*_ is determined by Eq. (4). Based on the above, a general formula in terms of the central wavelength *λ*, the DMD column number *n*, and the sCMOS column number *c* is established as indicated in Eq. (5), which is used for quantitative characterization of the law of the spectral shift on the sCMOS during the scanning process of the DMD.

1 $$c_{300} = \left\lceil {4.933\lambda - 1875.208} \right\rceil$$

2 $$c_n = \left\lceil {k_n\lambda + b_n} \right\rceil ,n \in \left[ {1,1024} \right]$$

3 $$\overline k = \frac{1}{{1024 - 70}}\mathop {\sum}\limits_{n = 1}^{1024 - 70} {k_n}$$

4 $$b_n = 0.7189n - 2115$$

5 $$c\left( {\lambda ,n} \right) = \left\lceil {5\lambda + 0.7189n - 2115} \right\rceil ,n \in \left[ {71,1024} \right]$$

Based on Eq. (5), supposing that the sample’s spectral range is from *λ*_1_ to *λ*_2_, the corresponding positions *c*_1_ and *c*_2_ on the sCMOS can be calculated by Eqs. (6) and (7). Clearly, the number of columns Δ*c* + 1 occupied by the sample’s spectra on each spectrally dispersed image is independent of the DMD column number *n*, and it will be a constant value once *λ*_1_ and *λ*_2_ are determined (Eq. (8)). The narrower the spectral range *λ*_2_–*λ*_1_ is, the smaller the number of columns Δ*c* + 1 is. Equation (8) is further rewritten as Eq. (9), and we can obtain the spectral sampling Δ*λ*_min_ = 0.2 nm when Δ*c*_min_ = 1. This means that the value of the spectral sampling is equal to the inverse of the mean value $\bar k$ of the dispersive equation’s slopes. Consequently, the number of spectral channels $\frac{{\lambda _{2\max } - \lambda _{1\min }}}{{\Delta \lambda _{\min }}} = 1,250$ is generated when *λ*_1 min_ = 450 nm and *λ*_2max_ = 700 nm.

6 $$c_1 = \left\lceil {5\lambda _1 + 0.7189n - 2115} \right\rceil$$

7 $$c_2 = \left\lceil {5\lambda _2 + 0.7189n - 2115} \right\rceil$$

8 $$\Delta c = c_2 - c_1 = \left\lceil {5\left( {\lambda _2 - \lambda _1} \right)} \right\rceil$$

9 $$\Delta \lambda = \lambda _2 - \lambda _1 = \frac{{\Delta c}}{5}$$

**Fig. 7:**
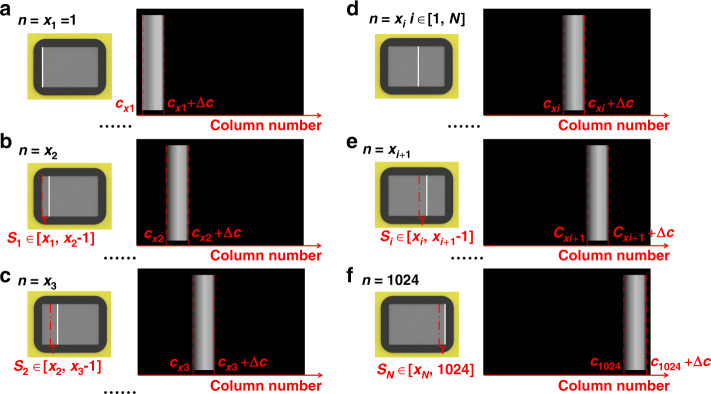
The sequential scanning of micromirrors from the first column to the end and their simultaneous recordings of spectrally dispersed images on the sCMOS. **a** The x1th-column micromirrors. **b** The x2th-column micromirrors. **c** The x3th-column micromirrors. **d** The xith-column micromirrors. **e** The xi+1th-column micromirrors. **f** The 1024th-column micromirrors.

**Fig. 8:**
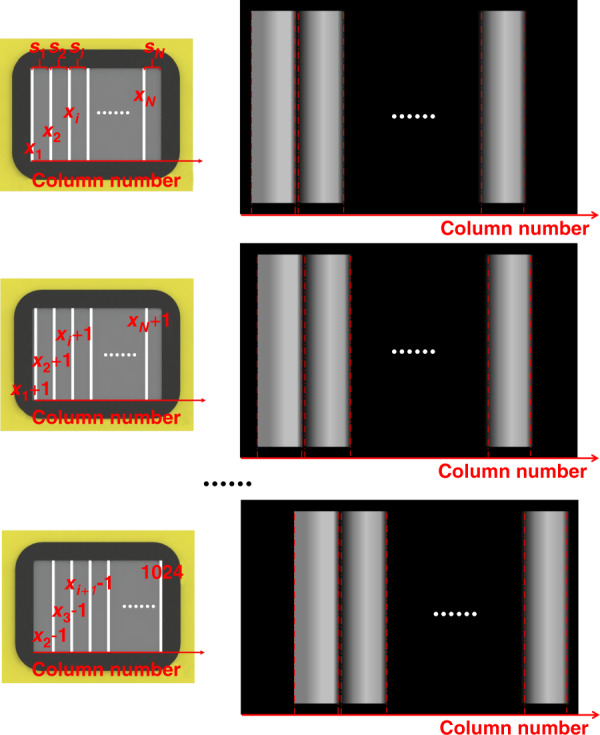
The parallel scanning of grouping micromirrors including *N* columns and the simultaneous recordings of spectrally dispersed images on the sCMOS

The DMD micromirrors are controlled to conduct sequential deflection line by line, resulting in the spectral shift on the sCMOS illustrated in Fig. 7. Accordingly, we study how to choose the number of DMD parallel scanning lines *N* (*N* > 1) and their corresponding scanning areas. As seen from Fig. 7, some spectra without overlap on the sCMOS may appear when *n* = *x*_*i*_, *i*∈[1, *N*]. This means that the *x*_*i*_th spectrum and the *x*_*i* + 1_th spectrum do not overlap in space if they are recorded simultaneously in a spectrally dispersed image by the sCMOS. As a result, to obtain high scanning efficiency, the DMD columns from *x*_1_ to *x*_*N*_ can be driven to perform the reflection together and then follow the DMD columns from *x*_1 _+ 1 to *x*_*N* _+ 1, as depicted in Fig. 8. Clearly, the narrower the spectral range is, the smaller the number of columns Δ*c* + 1 is. Note that the key point in performing multiline parallel scanning is to avoid spectral crosstalk between adjacent scanning groups. Consequently, the following conditions must be satisfied:

10 $$\left\{ {\begin{array}{*{20}{c}} {i \in \left[ {1,N} \right]} \\ {1 \le x_{i}\, <\, 1024} \\ {c_{xi + 1} \,> \, c_{xi} + \Delta c} \\ {c_{xi + 1} + \Delta c\, <\, 2048} \end{array}} \right.$$

where *c*_*xi*_ and *c*_*xi* _+ Δ*c* represent the number of sCMOS columns corresponding to the *x*_*i*_th DMD column covered by wavelengths *λ*_1_ and *λ*_2_, respectively; *c*_*xi* + 1_ and *c*_*xi* + 1 _+ Δ*c* represent the number of sCMOS columns corresponding to the *x*_*i* + 1_th DMD column covered by wavelengths *λ*_1_ and *λ*_2_, respectively.

By solving the above systems of inequalities, we can first define the number of parallel scanning lines *N* of the DMD and then estimate the data size and scanning time. If 1024 is divisible by *N*, each scanning area *s*_*i*_, the total scanning time *t*_*m*_, and the number of spectrally dispersed images *j*_*m*_ can be expressed by Eqs. (11), (12), and (13). Otherwise, they are represented as Eqs. (14), (15), and (16). When the DFMLPS-HSM system performs single-line scanning, it will take a scanning time of *t*_*s*_ = 1024*t* to accomplish the entire scanning process, and 1024 spectrally dispersed images will be acquired. Clearly, the higher the value of *N* is, the more dramatically the scanning time and the amount of data are reduced. It is important to note that the number of parallel scanning lines *N* of the DMD is determined only by the spectral range of the sample instead of any kind of imaging mode.

11 $$s_i \in \left[ {x_i,x_{i + 1} - 1} \right],i \in \left[ {1,N} \right]$$

12 $$t_m = \frac{{1024t}}{N}$$

13 $$j_m = \frac{{1024}}{N}$$

14 $$\left. {\begin{array}{*{20}{c}} {s_i \in \left[ {x_i,x_{i + 1} - 1} \right],i \in \left[ {1,N - 1} \right]} \\ {s_i \in \left[ {x_N,1024} \right],i = N} \end{array}} \right\}$$

15 $$t_m = t\left( {x_2 - x_1} \right),t_m \in \left( {\frac{{1024t}}{N},\frac{{1024t}}{{N - 1}}} \right)$$

16 $$j_m = x_2 - x_1,j_m \in \left( {\frac{{1024}}{N},\frac{{1024}}{{N - 1}}} \right)$$

where *t* is equal to the exposure time. Note that the exposure time *t* is not infinitely short and is determined by the relatively slow sCMOS, even though the DMD scanning process can be extremely fast. As the maximal frame rate of the sCMOS we used was 164 frames per second (fps), the minimum value of *t* was ~6 ms.

The implementation of controlling the DMD to perform multiline scanning can be mainly divided into three steps. First, control programs are created on the upper computer to generate the encoding patterns according to the number of DMD parallel scanning lines and their corresponding scanning areas. Each encoding pattern represents a scanning state where every micromirror should be either in the ‘on’ condition by being encoded as ‘1’ or in the ‘off’ condition by being encoded as ‘0’. When a micromirror is encoded as ‘1’, it will be electrically driven to be turned on and reflect the corresponding image to the spectral dispersion subsystem, and vice versa. Second, the programs are controlled to be uploaded onto an FPGA used as the DMD controller via USB high-speed transmission by the upper computer. In addition, some parameters, such as the exposure time and storage path, are set in the control software. Last, the FPGA controls the USB interface to receive the programs, and therefore, each micromirror is electrically driven to be turned on or off. Every time an encoding pattern is displayed steadily by the array of micromirrors, a synchronous signal from the DMD is sent to the sCMOS and triggers the sCMOS to record the corresponding spectrally dispersed image. After the spectrally dispersed image is received by the upper computer, the next encoding pattern will be loaded.
